# Wisdom in the digital age: a conceptual and practical framework for understanding and cultivating cyber-wisdom

**DOI:** 10.1007/s10676-022-09640-3

**Published:** 2022-03-04

**Authors:** Gianfranco Polizzi, Tom Harrison

**Affiliations:** Jubilee Centre for Character and Virtues, School of Education, University of Birmingham, Birmingham, B15 2TT UK

**Keywords:** Wisdom, Cyber-wisdom, Virtue ethics, The internet, Education

## Abstract

The internet presents not just opportunities but also risks that range, to name a few, from online abuse and misinformation to the polarisation of public debate. Given the increasingly digital nature of our societies, these risks make it essential for users to learn how to wisely use digital technologies as part of a more holistic approach to promoting human flourishing. However, insofar as they are exacerbated by both the affordances and the political economy of the internet, this article argues that a new understanding of wisdom that is germane to the digital age is needed. As a result, we propose a framework for conceptualising what we call *cyber-wisdom*, and how this can be cultivated via formal education, in ways that are grounded in neo-Aristotelian virtue ethics and that build on three prominent existing models of wisdom. The framework, according to which cyber-wisdom is crucial to navigating online risks and opportunities through the deployment of character virtues necessary for flourishing online, suggests that cyber-wisdom consists of four components: cyber-wisdom literacy, cyber-wisdom reasoning, cyber-wisdom self-reflection, cyber-wisdom motivation. Unlike the models on which it builds, the framework accounts for the specificity of the digital age and is both conceptual and practical. On the one hand, each component has conceptual implications for what it means to be wise in the digital age. On the other hand, informed by character education literature and practice, it has practical implications for how to cultivate cyber-wisdom in the classroom through teaching methods that match its different components.

## Introduction

We live in societies that are highly saturated with digital technologies. Since the advent of the internet, there have been growing concerns about the extent to which it presents not only opportunities but also risks that undermine human flourishing—our ability to live well and thrive both individually and collectively (Harrison, 2021; Jubilee Centre, 2017). On the one hand, the internet facilitates learning, socialisation and participation in society. It provides opportunities for boosting the economy, for connecting with different communities and for sharing public life. On the other hand, it exacerbates issues of privacy, financial safety, misinformation and, among other risks, forms of online abuse, such as cyberbullying (Livingstone et al., 2017).

The tension between online risks and opportunities raises ethical questions about how to use digital technologies responsibly, with users required to engage in processes of moral decision-making online; from observing copyright laws to navigating forms of incivility on social media platforms (see, for example, D’Errico & Paciello, 2018; Yoon, 2011). Moral decision-making is crucial both online and offline. However, what is unique about the digital age is that the risks that the internet presents—risks that raise challenges in terms of internet safety—are largely exacerbated by both its affordances (i.e., its technical features and what these do or do not enable users to do) and the nature of the digital environment (i.e., how internet corporations operate). Indeed, we are at a juncture in which public mistrust in the ability of tech companies to self-regulate the digital environment is rampant (Mansell, 2021). As a result, while both internet corporations and policymakers are confronted with the challenge of making ethical decisions about how the digital environment should be re-designed and managed (see, e.g., UK Government, 2020), users are presented with the task of navigating such an environment wisely—especially in the absence of meaningful regulation. This task is particularly important for users of all ages but, above all, for younger users, who are both the pioneers of and the most vulnerable in the digital age.

The challenges posed by the internet suggest that, in the digital age, wisdom—broadly understood as “the capacity to realize what is of value, for oneself and others” (Grossmann et al, 2020; Maxwell, 2007, p. 97)—is essential, on a macro level, as a collective endeavour that is necessary for re-imagining the digital environment and, on a micro level, as a quality that individuals and communities need online. Concerned with the latter, this article, which builds on recent renewed academic interest in the virtue of wisdom (e.g., Grossmann et al., 2020), considers the ways in which this virtue can be promoted, in the context of using the internet, via formal education. But what type of wisdom do users need in the digital age, and how can this be cultivated in practice in the classroom? This article argues that a novel understanding of wisdom is necessary in order to take into account both the affordances and the political economy of the internet. As a result, we propose in this article a framework for understanding what we call *cyber-wisdom* (i.e., the ability to do the right thing at the right time, when using the internet) and how this can be taught in practice through cyber-wisdom education (i.e., a form of character education, which is in turn a form of moral education, aimed at facilitating human flourishing online through the promotion of cyber-wisdom and character virtues) (Dennis & Harrison, 2020).

In order to develop our framework, we draw on three prominent models of wisdom as a multi-component construct that lie at the intersection of, but are primarily grounded in, different disciplines. More specifically, we take inspiration from Ardelt’s (2004) and Grossmann et al.’s (2020) models of wisdom, both of which stem from moral psychology, as well as from Darnell et al.’s (2019) and Kristjánsson et al.’s (2021) model of *phronesis* as practical wisdom, which is grounded in moral philosophy. While each of these models provides valuable insights into the nature of wisdom—insights that have implications for moral education—none of them examines the nature of wisdom in the digital age, nor how this should be cultivated in the classroom. By contrast, what distinguishes the framework that we propose by building on the models above is that it is (1) mindful of the nature of the internet and of the digital environment, and (2) not just conceptual, as in suggesting what wisdom consists of in the digital age, but also practical, as in drawing on character education literature and practice to reflect on how wisdom might be cultivated in practice in the classroom. Indeed, the idea behind these two interconnected aims is that we can only promote cyber-wisdom education provided we first understand what cyber-wisdom refers to as a concept.

The section below starts by discussing why we need wisdom in the digital age. Then, after introducing the concept of cyber-wisdom both in general and in the context of formal education, we review the three models of wisdom above. Building on these models, we propose a conceptual and practical framework for understanding and cultivating cyber-wisdom in ways that draw on character education literature and practice. Finally, we conclude with a section on the research and practical implications of the framework.

## Why we need wisdom in the digital age

Besides providing opportunities (e.g., for learning, socialisation and participation in society), the internet presents risks that undermine human flourishing, including, for example, online abuse, incivility, misinformation and polarisation. From a user perspective, the task of navigating both online risks and opportunities requires users to learn how to use the internet wisely and responsibly, which makes it imperative to cultivate wisdom as a character trait that is necessary for flourishing online. Such an imperative is made even more urgent by the fact that many online risks are exacerbated by (1) the affordances of the internet, and (2) how the digital environment operates and is (under-)regulated.

### The affordances of the internet

The notion of affordances, defined by Gibson (1979) as what the environment offers an individual, is often used within media studies to refer to what the technical features of digital technologies do or do not enable users to do (Hutchby, 2001). As suggested by Suler (2004), the internet is designed in ways that contribute to online opportunities and risks, as reflected in its implications for connectivity, proximity and anonymity. Arguably, one of the greatest advantages of using the internet is that it enables users to connect with one another both simultaneously and regardless of their location. While such a quality facilitates, especially when using social media platforms, opportunities for social interaction, networking and participation in public debate (see, for example, Jenkins et al., 2016), it also reduces distance between potential victims of online abuse and their perpetrators (Mishnan et al., 2009). This means, more concretely, that it contributes to online risks that can range from cyberbullying and online shaming to issues of trolling and incivility (Livingstone et al., 2017).

Because of limited visual cues and body language, which are typical of online communication, these forms of online abuse are amplified by the extent to which offenders can find it hard to reflect on the impact of, and accordingly change, their behaviour, be this intentional or inadvertent (Campbell, 2005). Relatedly, limited social presence can reduce their empathy and feelings of guilt (Malti et al., 2010). As a result, from the perspective of perpetrators, online abuse is often grounded in sentiments of hatred and a lack of sensitivity towards others (e.g., D’Errico & Paciello, 2018; Zezulka & Seigfried-Spellar, 2016). Meanwhile, it can have mental and emotional repercussions for victims, including feelings of fear, distress and anxiety (see, e.g., Ortega et al., 2012).

What is more, the internet makes it easier for users to interact with others anonymously. Such a quality enables users to avoid sharing their personal information when they may be subject to discrimination (Christopherson, 2007), but it also encourages them to feel more confident in posting negative comments on social media platforms (Suler, 2004). Furthermore, not only does it amplify the spread of misinformation, which in turn undermines citizens’ ability to engage in civil deliberation (Vaccarezza & Croce, 2021), but also exacerbates illegal activities, ranging from piracy and plagiarism to forms of online abuse, such as online grooming. These activities, again, have emotional consequences. Research shows that online piracy is often accompanied by emotions of shame and guilt (see, e.g., Herjanto, 2013). Meanwhile, the online grooming of children for sexual purposes is characterised by a push and pull on victims’ emotions, with sexual predators taking advantage of victims’ vulnerability by using charm to deceitfully express feelings of attachment to their victims (Whittle et al., 2015). This is why these issues, which raise the question of how to manage morally questionable behaviour online, suggest that wisdom is crucial for users to navigate online opportunities and risks that, amplified by digital affordances, are grounded in affect and reflect different perspectives, including those of victims and of perpetrators of online abuse.

### The digital environment

Besides its affordances, the internet contributes to online risks because of its political economy—that is, because of the ways in which internet corporations operate and are managed, which applies to the broader digital environment and relates to issues of internet governance. More specifically, the lack of meaningful internet regulation across Western countries exacerbates online risks that range from forms of online abuse and illegal activities to the polarisation of public debate and the spread of misinformation. This problem, in turn, makes it essential for users to rely on their own ability to navigate online risks and opportunities through making wise and responsible decisions. Historically, the internet was invented on libertarian principles of freedom, both in terms of freedom of expression online and in line with a free-market spirit that underpins how internet corporations like Google and Facebook operate (Dahlberg, 2010). This is why, for many decades, the governments of countries like the UK and the US have been reluctant to regulate the digital environment, conscious that attempts to do so are often ineffective and hard to implement. This is reflected, for instance, in the tension between observing copyright laws and accessing most information online free of charge, which generates, when it comes to piracy or plagiarism, feelings of anxiety among users as well as moral panics that legitimise interventions (see, e.g., Patry, 2009).

Recently, there have been growing concerns about internet corporations prioritising their economic interests—which rely on selling users’ data to advertising companies—in ways that invade users’ privacy and fuel online risks that hinder public debate (Hindman, 2018). Underpinned by sentiments of hatred and division, these risks range from trolling and online abuse by extremist groups motivated by racism to polarisation and misinformation that, capitalising on citizens’ feelings of distrust in and dissatisfaction with institutions, undermine democracy’s reliance on a well-informed citizenry (Vaidhyanathan, 2018). Indeed, internet corporations like Google and Facebook collect and track users’ data through algorithms that expose users to personalised content on the basis of how sensationalist this may be and in line with their pre-existing beliefs. To tackle this problem, policymakers within countries like the UK are now making active efforts to promote forms of regulation that problematise internet corporations’ attempts to self-regulate online content (see, e.g., UK Government, 2020). However, efforts to regulate the digital environment are patchy, are still in their infancy, and are bound to expose contradictions between the power of nation states and of big tech corporations operating globally (Reidenberg, 2005). As a result, especially in the absence of meaningful regulation, users are left with the challenging task of wisely navigating online opportunities and risks such as misinformation and polarisation, both of which deepen divisions by fuelling users’ fears and anxiety towards others (Vaidhyanathan, 2018).

## Cyber-wisdom: a conceptual and practical framework

As argued above, the internet presents not only opportunities but also risks, which undermine human flourishing and that are exacerbated by the affordances and the political economy of the internet. In this section, we define the concept of cyber-wisdom and provide a rationale as to why cyber-wisdom education is important for cultivating in users the ability to wisely navigate online risks and opportunities. Then, with a view to not just defining but also conceptualising cyber-wisdom, and how this can be taught in practice in the classroom as a multi-component construct, we review three models of wisdom that are prominent in the literature. Finally, while none of the models account for the specificity of the digital age, nor are particularly concerned with how wisdom can be taught in practice, we propose a framework for understanding and cultivating cyber-wisdom. On the one hand, the framework builds on three prominent models of wisdom. On the other hand, it is mindful of both the affordances and the political economy of the internet, and is not just conceptual but, informed by character education literature and practice, also practical.

Coined by Harrison (2016a, 2016b, 2021), the term cyber-*phronesis* (translated for practical use as “cyber-wisdom”) is grounded in Greek language and philosophy. Conceptually, it is inspired by the Aristotelian notion of *phronesis*. Etymologically, its prefix “cyber” derives from the term *kybernetes*, which 2000 years ago meant “helmsman” in Greek. The choice of this prefix is not random. The idea behind cyber-wisdom is that, like helmsmen, internet users are confronted today with the challenging task of navigating a complex environment that provides both opportunities and risks. This is why, in an age in which the internet has become so ubiquitous, it is imperative to equip children with the ability to make decisions online that are driven by virtues such as honesty and compassion and that enable them to choose the right course of action, especially when interacting and communicating with others. It is such an ability that can be defined as cyber-wisdom (Dennis & Harrison, 2020; Harrison, 2016a). Inspired by the Aristotelian intellectual quality of *phronesis*, cyber-wisdom serves as a meta-virtue that coordinates all other virtues online. It is the quality of being able to do the right thing, at the right time, when using the internet.

Conducted in the Jubilee Centre for Character and Virtues, Harrison’s work on cyber-wisdom is embedded in the long tradition of research that the Centre has undertaken throughout the years to promote character education—a form of moral education that it approaches in neo-Aristotelian virtue ethical terms. A few years ago, the Jubilee Centre (2017) updated its theoretical and practical framework for understanding and teaching character education. The framework suggests that virtues may be categorised as four different types—i.e., (1) intellectual (e.g., critical thinking), (2) moral (e.g., compassion), (3) civic (e.g., civility), and (4) performance (e.g., resilience)—with *phronesis* functioning as a meta-virtue moderating all other virtues. Applied to the digital age, cyber-wisdom education is an extension of the type of character education promoted by the Jubilee Centre. However, despite its importance, it has remained marginal within formal education, which is reflected in the extent to which its place in the school curriculum is both limited and unclear (Polizzi & Harrison, 2020).

In practice, and only to some extent, schools in the UK, for instance, teach elements of character and moral education in ways that overlap with digital citizenship education, which is concerned with the teaching of how to use digital technologies responsibly, especially in the context of interacting with others and participating in society (Ribble, 2007). However, neither character nor moral education nor digital citizenship education are firmly embedded in the national curriculum for England (Polizzi & Harrison, 2020). Elements of these forms of education are often taught at the discretion of schools through activities such as assemblies, days that are dedicated to raising awareness about the internet (e.g., Safer Internet Day), events and communications with parents, and through different subjects that range from Computing and Citizenship to Personal, Social, Health and Economics (PSHE) education. The curricula of these subjects, however, place little emphasis on the importance of developing both different virtues and wisdom as a meta-virtue. Rather, they privilege deontological and utilitarian approaches to moral education, approaches that encourage children, respectively, to follow rules and to reflect on the consequences of their online actions (Harrison, 2016b; Polizzi & Harrison, 2020).

Such approaches play a role in how students learn how to use the internet responsibly. However, we argue that they need to operate in tandem with a character-based approach, which is central to cyber-wisdom education, grounded in virtue ethics. Rules alone are not sufficient for guiding moral behaviour online. They can be too abstract, oblivious to the emotional aspects of moral motivation, and—as in the case of restrictions on screen time—inadequate for allowing children to build heathier relationships with digital technologies. Similarly, expecting children to reflect on the long-term implications of their online actions can be rather challenging and might work in some contexts but not in others (Dennis & Harrison, 2020, p. 5). This is why, besides following rules or evaluating the consequences of their own actions, children should be encouraged to develop different virtues such as honesty, compassion, generosity and respect for others—that is, habits of good action. But in order to coordinate which virtues users need to deploy online depending on context, which is particularly important when dealing with moral dilemmas that result from the clash of two or more virtues, they need cyber-wisdom. This may include, for example, deciding whether to protect, in the name of loyalty, a friend who perpetrates online abuse, or whether to report their behaviour in the name of honesty. In short, functioning as a meta-virtue, cyber-wisdom is the quality that can enable users to choose the right course of action through a process of intellectual reasoning that is contextually sensitive, and in ways that contribute to human flourishing.

But what does it mean to cultivate cyber-wisdom through formal education? To answer this question, we need to develop a more nuanced understanding of what cyber-wisdom refers to as a concept and how this can be taught in schools. In order to do so, in the next section we review three models of wisdom that are prominent in the literature. Then, we propose both a conceptual and a practical framework for understanding cyber-wisdom, and how this can be cultivated in practice in the classroom, by drawing on different aspects of these models in ways that are specific to the digital age.

### Three prominent models of wisdom

The last few decades have witnessed a rise in research on wisdom across different disciplines, including moral psychology (e.g., Baltes & Smith, 2008; Glück, 2009; Helson & Srivastava, 2002), moral education (e.g., Gregory, 2009; Huynh & Grossmann, 2020) and research informed by moral philosophy and, in particular, by neo-Aristotelian virtue ethics (e.g., Fowers, 2005; Kristjánsson et al., 2021; Schwartz & Sharpe, 2010). Back in the 1980s and 1990s, prior to this surge, some were shifting from researching wisdom, moral decision-making and moral behaviour as relying primarily on cognition—the mental structures of reasoning that are central to gaining knowledge and to making sense of the world (see, for example, Kohlberg, 1958)—to studying the process of making, and acting on, moral decisions as requiring additional components (e.g., Blasi, 1983; Montada, 1993, Narvaez & Rest, 1995). As such, throughout the years, both this process and the notion of wisdom have been approached as incorporating multiple dimensions, including meta-cognitive elements (i.e., higher order processes that enable individuals to evaluate their own cognitive processes) (e.g., Grossmann et al., 2020), affective aspects concerned with the deployment of feelings and emotions (e.g., Ardelt, 2004; Haidt, 2001), and elements of moral identity, understood as a source of moral motivation (e.g., Hardy & Carlo, 2005).

Such a more nuanced approach to the study of wisdom is better equipped to address some of the overarching questions that are relevant to wisdom researchers: what is wisdom? How is it developed? How does it manifest in the lived experiences of individuals? And how should it be conceptualised to bridge the gap between moral reasoning and moral action (i.e., the idea that one’s ability to make wise choices does not necessarily translate as acting wisely)? Indeed, identifying the right components of wisdom is key to answering these questions and to promoting wisdom through educational initiatives. But with the rise in research on wisdom as a multi-component construct, we have also witnessed both a rise in terminological confusion, with researchers from different disciplines using different terms to refer to similar components, and a proliferation of different approaches to wisdom that raise the question of which models of wisdom may be more helpful than others (Grossmann et al., 2020). As a result, while wisdom is essential in our complex and ever-changing world, wisdom researchers continue to grapple with fundamental questions.

This article proposes a framework that lays out the conceptual foundations of what wisdom refers to in the digital age, and how it should be cultivated in practice through formal education, which is a subject still in its infancy. In the sections that follow, we review three prominent models of wisdom, which are grounded primarily in moral psychology (i.e., Ardelt, 2004; and Grossmann et al., 2020) and in moral philosophy (i.e., Darnell et al., 2019; Kristjánsson et al., 2021). These are not the only models of wisdom that have been proposed in the wider literature (see, for example, Baltes & Staudinger, 2000; Stenberg & Karami, 2021),^1^ however, they are among the most comprehensive, which is ideal for capturing different nuances of wisdom and how this has been conceptualised across different disciplines. Since the concept of cyber-wisdom builds on neo-Aristotelian virtue ethics, one may think that the only model that has something to offer to the framework that we propose below is the one developed by Darnell et al. (2019) and Kristjánsson et al. (2021). This, however, is not the case, since each of the models incorporates aspects that are valuable for understanding the nature of wisdom in the digital age. On the one hand, the choice of these models is not representative of all the work that has been undertaken in this area. On the other hand, despite being oblivious to dynamics that characterise the digital age, the models below, which are reviewed in chronological order, offer valuable insights into the concept of wisdom and its development.

#### Ardelt’s model of wisdom

Developed in 2004 and grounded in moral psychology, Ardelt’s (2004) model of wisdom defines wisdom as incorporating three components: cognition, reflection and affect. With this model, Ardelt problematised the ways in which the concept had been approached by the Max Planck Institute (MPI) group in Berlin, one of the most prominent groups researching wisdom throughout the ‘90 s and early 2000s. According to the Berlin group, led by psychologist Paul Baltes, wisdom is, first and foremost, a collective product that is based on cognition but transcends the individual (Baltes & Staudinger, 2000). For Baltes and colleagues, it refers to “expert knowledge in the fundamental pragmatics of life that permits exceptional insight [and] judgement … about complex and uncertain matters” (Pasupathi et al., 2001, p. 351). This form of expertise, for the Berlin group, consists of cultural, “factual and procedural knowledge about the world” (Baltes & Staudinger, 2000, p. 76). This is why, according to Baltes and colleagues, wisdom is highly contextually dependent, which echoes the emphasis on social context typical of social psychology.

By contrast, Ardelt’s model frames wisdom as a personality trait that is embedded within the individual and relies not just on cognitive but also on meta-cognitive and affective processes. More specifically, for Ardelt, the cognitive dimension of wisdom, which consists of knowledge of human life and different events, is not sufficient in itself for individuals to be wise. Such a dimension is essential for identifying the ethical features of a given situation, which represents an ability that is often described within moral psychology as moral sensitivity (Rest, 1986). But according to Ardelt, a wise person needs to possess also the ability to examine their own motives and behaviour and to reflect on different events from multiple perspectives. Ardelt refers to such an ability as *reflection*, a form of meta-cognition. While cognition relies on knowledge that is more descriptive, reflection is based on interpretative knowledge, enabling individuals to develop “a deeper understanding of salient phenomena and events” (Ardelt, 2004, p. 262). For her, “reflective thought processes are essential to realize wisdom” (p. 268). But, as she postulates, a person can only be wise as long as they also deploy positive emotions such as sympathy and compassionate love for others—emotions that are crucial to the transcendence of one’s own subjectivity and to the realisation of one’s own motives, which lead, in turn, to moral action. This is why, as prescribed by her model, reflection (on one’s own personal experiences and on those of others) operates at the intersection of cognition and affect.

#### *Grossmann *et al*.’s common model of wisdom*

While Ardelt’s (2004) model dates back to a couple of decades ago, Grossmann et al.’s (2020)—referred to as the common model of wisdom—was developed recently on the basis of a survey that was administered among psychologists and moral scientists researching questions of wisdom and that culminated in a meeting with experts in the field. Through this survey, Grossmann and colleagues explored how researchers understand and operationalise the concept of wisdom with a view to proposing a model that captures common patterns. What emerged from their survey is that “moral aspirations and certain aspects of meta-cognition … [are] the most common features of the construct across definitions and operationalizations of wisdom” (p. 104).

Indeed, according to their model, wisdom can be understood as a two-component construct. First and foremost, it relies on aspects of meta-cognition that lie *de facto* at the intersection of cognition and meta-cognition. Grossmann and colleagues refer to these aspects as *perspectival meta-cognition* (PMC), which range from the ability to find a balance between different viewpoints and to know the limits of one’s own knowledge (which is what they describe as epistemic humility) to a disposition to consider multiple perspectives in ways that are contextually sensitive. These aspects, they argue, are particularly important within our contemporary societies, which are increasingly polarised. More specifically, they are crucial to navigating one’s own knowledge, to reflecting “on multiple issues at stake”, and to solving moral dilemmas involving different perspectives, as in the case of choosing between self-interests and collective interests (Grossmann et al., 2020, pp. 109–110). At the same time, according to their model, PMC is based on what they refer to as moral aspirations—ideals that constitute one’s own moral identity by providing the motivation needed to act wisely. Such ideals include, above all, an aspiration to pursue the truth, a shared sense of humanity, and an orientation towards the common good that relies on principles of in-group cooperation and pro-sociality.

The model proposed by Grossmann and colleagues is mindful that wisdom is contextually dependent and, while grounded in moral psychology, is informed to some extent by moral philosophy; in particular, by neo-Aristotelian virtue ethics and the notion of human flourishing. For them, PMC and moral aspirations operate in tandem with one another in ways that contribute, in line with ideals of the common good, to the ability to act on “character traits” such as epistemic humility—that is, on “psychological characteristics” or, in Aristotelian terms, *virtues* (Grossmann et al., 2020, p. 121).

#### *Darnell *et al*.’s and Kristjánsson *et al*.’s model of phronesis as practical wisdom*

While both Ardelt’s and Grossmann et al.’s models are rooted primarily in moral psychology, Darnell et al.’s (2019) and Kristjánsson et al.’s (2021) recently developed model of *phronesis* as practical wisdom, despite having multidisciplinary implications, is grounded primarily in moral philosophy and, in particular, in neo-Aristotelian virtue ethics. As explained earlier in this article, the Aristotelian concept of *phronesis*, or practical wisdom, functions as a meta-virtue that enables individuals, through a process of intellectual reasoning, to choose the right course of action. More precisely, it enables them to prioritise and act on different virtues (e.g., compassion, honesty). At the same time, it relies on context and aligns with the ultimate purpose of contributing to human flourishing (p. 4). Some have rejected the concept of *phronesis* as a meaningful and cohesive construct (see, e.g., Miller, 2021). For Lapsley (2019), for example, it is hard to translate such a philosophical concept into a psychological construct that incorporates elements that may be captured by a single measure. Lapsley (2019), furthermore, is sceptical about the extent to which moral identity, which often retains a dark side, may be reduced to an unequivocable positive in the context of exercising wisdom, or about the need to juxtapose cognition and affect, since the two are intrinsically intertwined. By contrast, conceptualised as a multi-component construct, *phronesis*, for Darnell, Kristjánsson and colleagues, relies on four distinct but interrelated components: (1) a constitutive function, which, similarly to the notion of moral sensitivity, refers to an understanding of the ethical aspects of different events, including what these require in terms of virtues; (2) an integrative function, which is central to moral reasoning—i.e., the ability to evaluate different events and choose the right course of action, especially when one is presented with moral dilemmas (e.g., to tell the truth to or hide it from a friend in the name of honesty or compassion, respectively); (3) emotional regulation, which refers to the ability to regulate one's own emotions in ways that align with an understanding of what the best course of action might be in a given context; and (4) a blueprint of the good life—that is, a motivation to deploy different virtues and to adjust one’s own identity in line with ideals of the common good.

These components, Darnell, Kristjánsson and colleagues argue, provide a richer understanding of what *phronesis* is as practical wisdom and have the potential to bridge the gap between moral reasoning and moral action. This is why, according to them, the concept incorporates not just cognitive and meta-cognitive elements (i.e., constitutive and integrative functions, respectively) but also affect (i.e., emotional regulation) as well as moral motivation/identity (i.e., blueprint). Strikingly, these components resonate to some extent with a model of virtue that was recently developed by Fowers et al. (2020). Informed by moral psychology and moral philosophy, this model, like the one proposed by Darnell et al. (2019) and Kristjánsson et al. (2021), is indebted to neo-Aristotelian virtue ethics. According to Fowers and colleagues, the concept of virtue relies on knowledge of context and of virtues, virtuous behaviour, emotions and a motivation to act on virtues as well as a dispositional aspect. To some extent, these elements echo Darnell et al.’s and Kristjánsson et al.’s components of *phronesis*, with virtuous knowledge and the emotional and motivational aspects of virtue overlapping, respectively, with the constitutive, emotional regulation and blueprint dimensions of practical wisdom. However, Fowers et al.’s model is limited to the concept of virtue. By contrast, Darnell et al.’s and Kristjánsson et al.’s model is concerned more comprehensively with wisdom as an overarching concept that, as explained above, is crucial to coordinating and deploying multiple virtues.

#### The three models in comparison

While Darnell et al.’s (2019) and Kristjánsson et al.’s (2021) model is arguably the most comprehensive, each of the models reviewed above provides valuable insights into the nature of wisdom. The models, however, are oblivious to whether and how the concept of wisdom should be understood and cultivated specifically in the digital age. Indeed, we live in an age that raises questions about how to develop and deploy wisdom from a user perspective in order to navigate online opportunities and risks. As examined earlier, these are amplified by the affordances and the political economy of the internet, from its technical features to the fact that it is under-regulated. Before discussing this further and addressing such a lacuna, Table 1 summarises what the models consist of and how they differ from one another.

**Table 1:** Three prominent models of wisdom as a multi-component construct

Ardelt (2004)—wisdom	Grossmann et al. (2020)—common wisdom	Darnell et al. (2019) and Kristjánsson et al. (2021)—*phronesis* (practical wisdom)
1. Cognition *Description:* Knowledge of human life and of the ethical features of different events	1. Perspectival meta-cognition (PMC) *Description:* Meta-cognitive processes necessary to navigate knowledge of different events and their ethical features, moral dilemmas and multiple perspectives—i.e., (1) balance of viewpoints, (2) epistemic humility, (3) context adaptability, (4) multiple perspectives	1. Constitutive function *Description.:* Knowledge of the ethical features of different events and what virtues apply to those events
2. Reflection *Description:* Self-examination and examination of events from multiple perspectives		2. Integrative function *Description:* Evaluation of events, particularly when these present moral dilemmas that depend on context
3. Affective *Description:* Deployment of positive emotions towards others (e.g., compassion, sympathy)		3. Emotional regulation *Description:* Ability to regulate one’s own (positive and negative) emotions in line with an understanding of what the best course of action might be in a given context
	2. Moral aspirations *Description:* Moral grounding of meta-cognition—i.e., (1) pursuit of truth and (2) common good orientation (shared humanity, balance of self-protection and orientation towards others, pro-sociality, virtuousness, in-group cooperation)	4. Blueprint *Description:* Motivation to deploy different virtues and to adjust one’s own identity in line with ideals of the common good

As argued above and captured by Table 1:

(i) While Ardelt’s (2004) model frames wisdom primarily as a quality of the individual with little attention to the social context, Grossmann et al.’s (2020) model as well as Darnell et al.’s (2019) and Kristjánsson et al.’s (2021) model of *phronesis* place more emphasis on the role of wisdom as contextually dependent.
(ii) Both for Ardelt and for Darnell, Kristjánsson and colleagues, cognition and meta-cognition represent distinct components of wisdom (referred to, respectively, as “cognition” and “reflection” by Ardelt, and as “constitutive” and “integrative” by Darnell et al. and Kristjánsson et al.). By contrast, Grossmann et al. place more emphasis on the importance of perspectival meta-cognition (i.e., “PMC”) as a component that lies *de facto* at the intersection of cognition and meta-cognition. For them, PMC is essential for navigating both multiple perspectives, which applies also to Ardelt’s component of reflection, and, relatedly, moral dilemmas involving different perspectives.
(iii) Unlike Ardelt’s model, Grossmann et al.’s model as well as Darnell et al.’s and Kristjánsson et al.’s model include moral identity/motivation as a crucial dimension of wisdom (referred to as “moral aspirations” and “blueprint”, respectively).
(iv) Unlike Grossmann et al.’s model, both Ardelt’s model and the one developed by Darnell et al. and Kristjánsson et al. emphasise the importance of, on the one hand, cognition and meta-cognition and, on the other, affect. It is important to note that Grossmann and colleagues have argued that emotional regulation may contribute to the development of wisdom (see, for example, Grossmann et al., 2019). However, according to them, it does not necessarily guarantee such development, which is why it is not constitutive of wisdom, as reflected in their model. Meanwhile, the affective component of Ardelt’s model, unlike the one proposed by Darnell, Kristjánsson and colleagues, does not concern emotional regulation *per se* (i.e., the ability to regulate one’s own positive *and* negative emotions), but, more simply, the ability to deploy positive emotions for moral purposes (e.g., compassion, sympathy).
(v) Ardelt’s model does not draw on virtue ethics, even though its affective component requires the deployment of positive emotions that resonate with virtues such as compassion and sympathy. By contrast, Grossmann et al.’s model as well as Darnell et al.’s and Kristjánsson et al.’s model are respectively informed by and grounded in neo-Aristotelian virtue ethics.

Clearly, all these models offer valuable insights into how the concept of wisdom can be understood. However, they pay little attention to the extent to which we live in an age that is highly mediated by digital technologies, and to what this means for how wisdom may be understood and cultivated, all of which raises the following questions:

(vi) Both for Ardelt and for Darnell, Kristjánsson and colleagues, cognition is crucial to understanding different events and their ethical implications. But what does it mean to understand the ethical features that are inherent in online opportunities and risks? Relatedly, what should users know in order to navigate both opportunities and risks in ways that are underpinned by an understanding of how different virtues apply to online, and therefore in ways that may differ from, offline contexts?
(vii) Grossmann et al.’s meta-cognitive elements of wisdom, as well as Darnell et al.’s and Kristjánsson et al.’s integrative function of *phronesis*, suggest that individuals should use reasoning to choose the right course of action, especially when presented with moral dilemmas. But how should users navigate moral dilemmas online that may be particular to the digital age because of the affordances and the political economy of the internet? Relatedly, what should users understand about the ways in which different virtues can clash online, which may raise tensions that might not necessarily apply to offline contexts?
(viii) According to Grossmann and colleagues, wisdom is essential in our increasingly polarised world, which is why meta-cognition for them, like reflection for Ardelt, is crucial to navigating multiple perspectives. But how can users navigate multiple perspectives online when the affordances and the political economy of the internet exacerbate the extent to which polarisation in the digital age fuels and builds on emotions, including sentiments of hatred towards others? Meanwhile, how can we draw on Darnell et al.’s and Kristjánsson et al.’s component of emotional regulation in the context of navigating different perspectives when their component is primarily concerned with regulating one’s own emotions rather than navigating those of others?
(ix) Grossmann et al. as well as Darnell et al. and Kristjánsson et al. understand wisdom as incorporating moral aspirations that guide moral behaviour in line with ideals of the common good. But what type of expectations should users have of the digital age, of the ways in which users interact with one another, and of how the digital environment is designed and managed?

It is with these questions in mind that in the next section we build on the models above to propose a framework for understanding cyber-wisdom as a multi-component construct, and for how this can be cultivated in practice through formal education.

### A framework for understanding cyber-wisdom, and how this can be cultivated in practice, as a multi-component construct

The three prominent models reviewed above are concerned with wisdom, but not in relation to using digital technologies. Not only does this lacuna justify the need for the framework that we propose below, but such a need is made even more urgent by the extent to which empirical research concerned with digital technologies has under-explored the concept of wisdom. Promisingly, a few studies at the intersection of media studies and moral psychology have focused on moral decision-making online by examining, for instance, the extent to which users value good character or are conscious of and respond to moral dilemmas, relating, for example, to online privacy and security (Jackson et al., 2008; Mohammadnazar et al., 2019). In addition, there is some research that has examined the extent to which low moral sensitivity corresponds to abusive behaviour online (Ge, 2020; Zezulka & Seigfried-Spellar, 2016). These studies have prioritised aspects that are relevant to wisdom in the digital age—aspects that range from how users deal with moral dilemmas to their moral sensitivity. However, they have under-explored wisdom as an overarching concept or how this can be understood or cultivated in practice.

This section therefore proposes a framework that aspires to conceptualise cyber-wisdom, and how this can be taught in the classroom, as a multi-component construct. While both the conceptual and practical dimensions of each component of cyber-wisdom are summarised in Table 2, in the text that follows we unpack the conceptual dimension of each component by building on the models reviewed above in ways that are mindful of the nature of the digital age. At the same time, we draw on character education literature and practice—and, in particular, on the work of the Jubilee Centre (2017) in relation to different aspects relating to the concept of virtue—in order to reflect on the practical dimension of each component. In doing so, we focus on how cyber-wisdom may be taught in schools through different teaching delivery methods that match its different components. In other words, within each section below, we first focus on the conceptual make-up of each component of cyber-wisdom by drawing on the models reviewed earlier in this article. Then, we draw on character education literature and practice to provide some directions as to how different teaching delivery methods could be used in the classroom across different key stages in order to teach each component of cyber-wisdom.

**Table 2:** Cyber-wisdom, and how it can be taught in practice, as a multi-component construct

Component	Description	Builds on models of wisdom	Builds on character education literature and practice
1. Cyber-wisdom literacy	Understanding of the nature of different virtues such as compassion and honesty as well as of the contexts and ways in which different virtues apply to, and can be deployed in, the digital age	• Ardelt’s (2004) wisdom component of cognition (knowledge of human life and events) • Grossmann et al.’s (2020) wisdom component of PMC (meta-cognition necessary to navigate knowledge of different events) • Darnell et al.’s (2019) and Kristjánsson et al.’s (2021) constitutive function of *phronesis* (understanding of different events and what virtues apply to those events)	• Jubilee Centre’s (2017) concept of virtue literacy (knowledge of different virtues and when these may be needed) To be taught through: • Narratives and stories (see, e.g., Arthur et al., 2014a; Carr & Harrison, 2015) aimed at encouraging students to develop an understanding of what different virtues entail and how these apply to, and can be deployed within, online contexts
2. Cyber-wisdom reasoning	Evaluation of, and ability to prioritise, different virtues online, particularly when these clash depending on context	• Grossmann et al.’s (2020) wisdom component of PMC (meta-cognition necessary to navigate dilemmas) • Darnell et al.’s (2019) and Kristjánsson et al.’s (2021) integrative function of *phronesis* (evaluation of events that present dilemmas)	• Jubilee Centre’s (2017) concept of virtue reasoning (deliberation aimed at choosing which virtues to deploy depending on context) To be taught through: • Classroom discussions (see, e.g., Harrison et al., 2018; Hedayati-Mehdiabadi et al., 2020) aimed at encouraging students to evaluate possible dilemmas online both hypothetically and in relation to their own online experiences
3. Cyber-wisdom self-reflection	Reflection on the moral dimensions of one’s own experiences online in ways that are grounded in (1) awareness of one’s own biases and how these can clash with the perspectives of others, and (2) the ability (a) to regulate one’s own emotions (e.g., when experiencing ethical dilemmas online) and (b) to navigate, depending on context, the emotions of others	• Ardelt’s (2004) wisdom component of reflection (self-examination of events from multiple perspectives) • Grossmann et al.’s (2020) wisdom component of PMC (meta-cognition necessary to navigate multiple perspectives) • Darnell et al.’s (2019) and Kristjánsson et al.’s (2021) emotional regulation component of *phronesis* (ability to regulate one’s own emotions in line with an understanding of what the best course of action might be in a given context)	• Jubilee Centre’s (2017) concept of virtue emotion (navigating emotions associated with different virtues) To be taught through: • Journals and diaries (see, e.g., Arthur et al., 2014b; Arthur et al., 2016) aimed at encouraging students to write about and reflect on their online experiences, the extent to which these are driven by emotions, and how the latter can be regulated in ways that are mindful of one’s own biases as well as the perspectives and emotions of other users
4. Cyber-wisdom motivation	Desire to act online on different virtues in line with a vision of the digital world that is underpinned by principles of the common good	• Grossmann et al.’s (2020) wisdom component of moral aspirations (common good orientation) • Darnell et al.’s (2019) and Kristjánsson et al.’s (2021) blueprint component of *phronesis* (motivation necessary to adjust one’s own identity in line with their vision of the common good)	• Jubilee Centre’s (2017) concepts of virtue identity (a commitment to deploying virtues) and virtue motivation (a desire to act on virtues) To be taught through: • Stories and discussions about exemplars and role models (see. e.g., Zagzebski, 2017) aimed at encouraging students to develop and draw on their moral motivation within online contexts

There is growing interest among researchers of moral and character education in how wisdom should be taught in schools, with some emphasis on the role of educators, teaching materials and different learning environments in the context of promoting wisdom through formal education (see, e.g., Huynh & Grossmann, 2020). It should be clarified, however, that the lack of a cohesive approach to wisdom as a multi-component construct makes it challenging to identify how to teach it in practice. Furthermore, research and practice promoting not just wisdom but, more specifically, cyber-wisdom education is still in infancy. Indeed, besides a school intervention that is currently being rolled out by the Jubilee Centre (2021) with a view to evaluating the effectiveness of a cyber-wisdom education programme across secondary schools in England, the notion of cyber-wisdom, as discussed earlier, is marginal within formal education, as is its overlap with digital citizenship education (Harrison & Polizzi, 2021; Polizzi & Harrison, 2020). Designed and evaluated by the Jubilee Centre, the school intervention that is currently being implemented in England is one of the very first attempts to measure the effectiveness of a school programme that aims at cultivating the different components of cyber-wisdom among 13–16-year-old students. As a result, when it comes to the practical dimension of each component of cyber-wisdom, what follows in this section is simply a sketch of how cyber-wisdom could be taught in the classroom as a multi-component construct. Informed by character education literature and practice, our suggestions below, although preliminary at this stage, resonate with the format used for the cyber-wisdom education programme.

#### Cyber-wisdom literacy

Embedded primarily within cognition, this component refers to an understanding of different virtues such as compassion and honesty as well as of the contexts and ways in which such virtues apply to, and can be deployed in, the digital age. To a large extent, the implications for wisdom inherent in this component resonate (1) with Ardelt’s (2004) component of cognition, which consists of knowledge of human life and events, (2) with Grossmann et al.’s (2020) component of PMC, particularly in relation to the role that cognition plays, in the form of knowledge of different events, as the foundation of the meta-cognitive processes that are crucial to navigating such knowledge, and (3) with Darnell et al.’s (2019) and Kristjánsson et al.’s (2021) constitutive function of *phronesis*, which requires the use of cognition for understanding what virtues apply to different events. Unlike these models, however, cyber-wisdom literacy is concerned specifically with the digital age. As argued above, the internet amplifies online risks, including, for instance, online abuse and misinformation, because of its affordances (e.g., in terms of connectivity and anonymity), and because of the libertarian ethos that underpins the extent to which search engines and online platforms are under-regulated.

However, the internet is also a force for good that presents considerable advantages (e.g., for learning, socialisation and participation). As a result, on the one hand cyber-wisdom literacy requires an understanding of the virtues that are relevant to different online contexts. This may include, for instance, appreciating the importance of showing compassion to users who receive negative comments on social media, or of producing and disseminating online one’s own views in line with principles of integrity and respect for others. On the other hand, such an understanding needs to be mindful of the ways in which different virtues can be deployed in ways that maximise online opportunities while minimising online risks. That is, possessing cyber-wisdom literacy means to understand not only 1) the nature of multiple virtues online, but also 2) the ethical dimensions of online opportunities and risks and, ultimately, 3) how multiple virtues can be acted upon in ways that preserve a balance between taking advantage of online opportunities and avoiding or coping with online risks. This may include, for example, understanding the value of accessing a wide range of information online in ways that are underpinned by virtuous curiosity, while reducing the spread of online misinformation, which is amplified by digital technologies, by sharing or producing content in ways that are honest.

Teaching cyber-wisdom literacy could rely, for instance, on the use of narratives and stories, so as to encourage students to develop an understanding of the role of different virtues online. As such, this component resonates with how the concept of virtue literacy (i.e., knowledge and understanding of different virtues, and of when these may be needed) may be taught in the classroom, according to the Jubilee Centre (2017). Indeed, as in the case of virtue literacy, which is an aspect that is central to the concept of virtue, the benefits of this method for teaching moral character are well-documented (see, for example, Arthur et al., 2014a; Carr & Harrison, 2015). This means that teachers could use, for example, real stories of virtuous practice based on online opportunities (e.g., online communities working together in the name of solidarity) as well as stories of online abuse, or of other online risks such as plagiarism, in order to teach students about the importance of possessing and showing online different virtues, from empathy and compassion to honesty and respect for others. Relatedly, what this also means is that cyber-wisdom literacy could be taught alongside the teaching of digital literacy, understood not just as practical digital skills and the critical ability to evaluate online content, but also as an understanding of the digital environment, along with the advantages and disadvantages that the internet presents both in general and for civic life (Polizzi, 2020, 2021).

#### Cyber-wisdom reasoning

This component, which is grounded in meta-cognition, refers to the ability to evaluate and prioritise different virtues online, especially when these clash depending on context. As such, it builds (1) on Grossmann et al.’s (2020) component of PMC, particularly in relation to its importance for navigating moral dilemmas, and (2) on Darnell et al.’s (2019) and Kristjánsson et al.’s (2021) integrative function of *phronesis*, which is concerned with the evaluation of events, especially when these present moral dilemmas that are based on the clash of two or more virtues. However, unlike their models of wisdom, cyber-wisdom reasoning is grounded in the recognition that moral dilemmas online may be exacerbated both by the affordances and by the political economy of the internet. Examples of such dilemmas may include accessing information free of charge versus observing copyright laws, which may be particularly constraining for users with limited financial resources, or whether or not to show respect, or even compassion, for users who show abusive traits on platforms like Facebook.

Social media platforms afford users the ability to disguise their identities more easily, to post negative comments with little scrutiny, and to respond to online abuse with limited repercussions beyond blocking or reporting users. Possessing cyber-wisdom reasoning relies therefore on the ability to (1) deal with the ethical implications of online contexts, (2) choose between multiple virtues online, and (3) factor in whether and how experiencing moral dilemmas online may involve scenarios that are specific to using the internet. This means that users can only exercise cyber-wisdom reasoning as long as they account for the ways in which dealing with moral dilemmas may be different online than offline. This, in turn, suggests that drawing on past experiences offline may be helpful for making informed decisions online only to a limited extent. In other words, in order to deploy cyber-wisdom reasoning, users need to draw primarily on their experience of using digital technologies, while adapting to the extent to which these are constantly evolving, especially in terms of their affordances.

A useful way to teach cyber-wisdom reasoning could be to have classroom discussions aimed at encouraging students to evaluate online dilemmas both hypothetically and, in ways that tap into their experiences of informal learning, in relation to their own online engagement beyond the classroom. Conceived in this way, this component echoes how the concept of virtue reasoning (i.e., deliberation aimed at choosing which virtues to deploy depending on context) may be cultivated in practice via formal education (Jubilee Centre, 2017). Indeed, we know from character education research that asking students to explore and discuss ethical dilemmas contributes to their ability to use moral reasoning to evaluate multiple scenarios and choose the best course of action in a given situation (Harrison et al., 2018; Hedayati-Mehdiabadi et al., 2020).

#### Cyber-wisdom self-reflection

This is the component of cyber-wisdom that is most distinct about our framework. Lying at the intersection of meta-cognition and affect, cyber-wisdom self-reflection refers to the ability to reflect on the moral dimensions of one’s own experiences online in ways that are grounded in (1) awareness of one’s own biases and how these can clash with the perspectives of others, and (2) the ability (a) to regulate one’s own emotions (e.g., when experiencing moral dilemmas online) and (b) to navigate, depending on context, the emotions of others. Conceived as such, the first aspect of this component builds on Ardelt’s (2004) component of reflection, understood as the self-examination of events from multiple perspectives, which applies also to Grossmann et al.’s (2020) component of PMC. Meanwhile, the second aspect of this component builds on Darnell et al.’s (2019) and Kristjánsson et al.’s (2021) emotional regulation component of *phronesis*, which refers to the ability to regulate one’s own emotions in line with an understanding of what the best course of action might be in a given context. However, whilst Ardelt’s model is valuable for conceiving of reflection as a form of meta-cognition that sits between cognition and affect, her affective component is limited to the ability to deploy positive emotions for moral purposes. By contrast, while Grossmann et al.’s model is oblivious to affect, cyber-wisdom self-reflection, like Darnell et al.’s and Kristjánsson et al.’s component of emotional regulation, is concerned with the process of navigating both positive and negative emotions. At the same time, it suggests that such a process, when it comes to using digital technologies, needs to operate in tandem with the task of understanding multiple perspectives.

This is because, as argued earlier in this article, both the affordances of the internet and the ways in which internet corporations operate exacerbate online risks that build on and fuel different perspectives and emotions among individuals as well as communities. Such emotions may include, for instance, hatred and distress, underpinning, respectively, perpetrators’ and victims’ experiences of online abuse as well as fear towards others and distrust in institutions fuelling both the polarisation of public debate and spread of misinformation online. As a result, this component, which is mindful of the nature of the digital age, prescribes that users need to be able to (1) reflect on their own biases and regulate their own emotions when dealing with moral dilemmas online (e.g., when managing feelings of empathy or anger in the context of interacting with users who show abusive traits), and, at the same time, (2) navigate the emotions of others within online settings in which their own biases might clash with the perspectives of others (e.g., within online contexts of public debate affected by polarisation fuelled by sentiments of hatred).

Cyber-wisdom self-reflection could be taught by asking students to keep journals and diaries, which is a method that is particularly valuable for encouraging students to develop character through self-reflection on their own practices and experiences (Arthur et al., 2016). As such, this component resonates with how the concept of virtue emotions (i.e., the practice of navigating emotions associated with different virtues) may be taught in the classroom, as discussed by the Jubilee Centre (2017). More specifically, in order to cultivate this component of cyber-wisdom, students could be asked to write about and reflect on the moral implications of their online experiences, on the extent to which these are driven by different emotions, and whether, and if so how, they manage to regulate their emotions in ways that are mindful of their own biases as well as of the perspectives and emotions of other users.

#### Cyber-wisdom motivation

This component refers to a desire to act online on different virtues in line with ideals of the digital world that are underpinned by principles of the common good. Defined as such, it builds on (1) Grossmann et al.’s (2020) component of moral aspirations, particularly when understood as an orientation towards the common good, and (2) Darnell et al.’s (2019) and Kristjánsson et al.’s (2021) blueprint component of *phronesis*, which consists of the motivation necessary to adjust one’s own identity in line with ideals of the common good. Unlike their models of wisdom, however, this component is concerned with the digital age. That is, it is mindful of the affordances and the political economy of the internet, which, as discussed above, exacerbate online risks such as online abuse and polarisation. This means that users’ moral aspirations could include, for instance, expecting users to interact online in honest and compassionate ways, expecting online communities to engage in public debate in ways that enable them to voice their own different concerns while also respecting a certain degree of civility (which is crucial to the functioning of democracy), or expecting internet corporations and policymakers to make more efforts to tackle online risks in line with virtuous principles of transparency, accountability and social justice. In short, cyber-wisdom motivation is crucial to the moral identity of users insofar as it provides them with a set of aspirations that are important to their own sense of self and that operate in tandem with the other components of cyber-wisdom. Such aspirations are essential for guiding users’ moral behaviour on the internet in line with normative ideals of how this should be used from a user perspective and managed collectively to facilitate human flourishing.

With this in mind, cyber-wisdom motivation could be taught through the use of stories and discussions about exemplars and role models aimed at encouraging students both to develop, through admiration and emulation, and to deploy moral aspirations that relate to the internet and apply to different online contexts. Conceived in this way, this component echoes how the concepts of virtue identity (i.e., a commitment to deploying virtues) and of virtue motivation (i.e., a desire to act on virtues) may be taught in practice in the classroom (Jubilee Centre, 2017). Indeed, the benefits of using this teaching method for promoting character and moral education are well-established (see, e.g., Zagzebski, 2017). This means that teachers could draw, for instance, on exemplars of online activism who have been committed to campaigning against cyberbullying, including activists such as Lizzie Velasquez.

## Conclusions

This article proposes a framework for understanding wisdom, and how this can be cultivated via formal education, in the digital age. The internet presents not just opportunities but also risks, which undermine human flourishing and make it essential for users to use the internet wisely and responsibly. Amplified by the affordances and the political economy of the internet, these risks build on and fuel a range of different perspectives and emotions—from hatred and distress, respectively underpinning perpetrators’ and victims’ experiences of online abuse, to fear towards others and distrust in institutions, feeding, in turn, into polarisation and the spread of misinformation online. After reviewing three prominent models of wisdom that are grounded primarily in moral psychology (i.e., Ardelt, 2004; Grossmann et al., 2020) and in moral philosophy (i.e., Darnell et al., 2019; Kristjánsson et al.'s, 2021), this article has proposed a new framework for understanding and promoting cyber-wisdom as a four-component construct. Each component has both conceptual and practical implications. Conceptually, it has implications for understanding wisdom in ways that build on the models above but apply specifically to the digital age. Practically, informed by character education literature and practice, it suggests how cyber-wisdom can be taught in the classroom through different teaching methods that are appropriate to its different components.

Indebted to neo-Aristotelian virtue ethics, our framework suggests that cyber-wisdom—the quality of being able to do the right thing, at the right time when using the internet—functions as a meta-virtue that coordinates all other virtues. Meanwhile, informed by the models above, it suggests that cyber-wisdom should incorporate four components: (1) cyber-wisdom literacy, (2) cyber-wisdom reasoning, (3) cyber-wisdom self-reflection, and (4) cyber-wisdom motivation. According to the framework, cyber-wisdom literacy, which requires an understanding of the nature of different virtues within online contexts, may be cultivated in the classroom through narratives and stories. Cyber-wisdom reasoning, which may be taught though the use of classroom discussions, relies on the ability to evaluate and prioritise different virtues online, particularly when these clash depending on context. Cyber-wisdom self-reflection, which may be developed by encouraging students to keep journals and diaries, consists of reflection on the moral dimensions of one’s own experiences online. This type of reflection is grounded both in awareness of one’s own biases vis-à-vis the perspectives of others, and in the ability to regulate one’s own emotions and to navigate those of others. Finally, cyber-wisdom motivation, which may be cultivated in schools through the use of stories and discussions about exemplars and role models, refers to a desire to act online on different virtues in line with ideals of the common good.

On the one hand, these four components of cyber-wisdom differ from the models above insofar as they are concerned with the use of digital technologies—and, more specifically, with navigating both online opportunities and risks. On the other hand, a particularly distinct feature of our framework lies in its component of cyber-wisdom self-reflection, which sits between meta-cognition and emotional regulation. Inasmuch as the internet exacerbates online risks that are deeply grounded in affect, the framework suggests that users need not only to reflect on their own biases and on the perspectives of others, but also to regulate their own emotions (e.g., when experiencing moral dilemmas online) as well as those of others in ways that are contextually sensitive.

In terms of future directions, the framework has both research and practical implications. First of all, while it requires empirical testing across different educational contexts and among different age groups, it invites wisdom researchers both within moral psychology and within moral philosophy to consider more closely whether, to what extent and in what ways meta-cognition and affect are intertwined within processes of moral decision-making online. Second, it invites researchers of moral education to focus more closely on how wisdom should be cultivated via formal education in the digital age. Third, it has implications for research on digital citizenship education. As argued above, this is concerned with the teaching of how to use digital technologies responsibly, which is why more research is needed on its intersection with cyber-wisdom education (see, for example, Harrison & Polizzi, 2021).

In terms of practical implications, the framework invites educationalists and policymakers within different countries to promote cyber-wisdom and its four components via national curricula. Relatedly, it suggests that teaching resources and teacher training should also be designed in order to support educators in cultivating cyber-wisdom. Finally, while this article is primarily concerned with the extent to which users engage wisely with digital technologies, and with the role of educators in shaping students’ online engagement, the framework proposed here should be promoted as part of a more holistic approach to re-imagining the digital environment. Such an approach requires different actors—including, besides educators, parents as well as governments and internet corporations—to share responsibility in terms of not just educating users but also redesigning and better regulating the digital environment. This is why further research is needed on the ways in which the framework presented in this article does not just promote human flourishing online by placing responsibility on users and educators, but does so as part of a collective endeavour.

## Footnotes

1 Arguably, Stenberg and Karami’s (2021) proposed unified framework functions more as a lens through which to review and evaluate theories of wisdom, rather than as an actual model of wisdom as a multi-component construct.
